# [Expression of Concern] Suppression of PTEN/AKT signaling decreases the expression of TUBB3 and TOP2A with subsequent inhibition of cell growth and induction of apoptosis in human breast cancer MCF-7 cells via ATP and caspase-3 signaling pathways

**DOI:** 10.3892/or.2026.9179

**Published:** 2026-08-12

**Authors:** Zhenhua Yang, Ying Liu, Changzheng Shi, Yuqin Zhang, Rongzhao Lv, Rong Zhang, Qian Wang, Yiming Wang

Oncol Rep 37: 1011–1019, 2017; DOI: 10.3892/or.2017.5358

Following the publication of the above article, a concerned author drew to the authors' attention that, regarding the data showing caspase-3 activity as measured by a colorimetric assay in Fig. 7 on p. 1015, given the use of the MCF-7 cell line in this study, it was unexpected that the authors would have identified changes in caspase-3 activity, since it has been demonstrated that MCF-7 cells do not express caspase-3 [for example, see the paper by Jänicke and colleagues entitled ‘MCF-7 breast carcinoma cells do not express caspase-3’. Breast Cancer Res. Treat., vol. 117.1 (2009); p. 219–221]. In addition, the reader noted that, in the Materials and methods section, it was stated that an Ac-DEVD-pNA-based assay had been used, which has been reported not to be specific for caspase-3, since this assay also reacts to caspase-7 [‘Preparation of the caspase-3/-7 substrate Ac-DEVD-pNA via solution-phase peptide synthesis’. Nat Protoc., vol. 5(2) (2010); p. 294–302]. The reader also noted that it was surprising that none of the western blot experiments included in this study actually probed for the presence of the caspase-3 protein, given that the caspase-3 signaling pathway represented one of the principal foci of the study.

The authors have been contacted by the Editorial Office to offer an explanation for these apparent anomalies or curiosities in the presentation of the data in this paper, and we are awaiting their response. Owing to the fact that the Editorial Office has been made aware of potential issues surrounding the scientific integrity of this paper, we are issuing an Expression of Concern to notify readers of this potential problem while the Editorial Office continues to investigate this matter further.

